# Development and explanation of electrocardiogram-based deep learning for predicting short-term mortality in heart failure patients

**DOI:** 10.7189/jogh.16.04048

**Published:** 2026-02-13

**Authors:** Yan Li, Lixia Cheng, Hongmin Zhou, Hongwei Yu, Zeyuan Liu, Qiuju Zhang

**Affiliations:** 1 Department of Biostatistics, Harbin Medical University, Harbin, China

## Abstract

**Background:**

Heart failure mortality has risen sharply after years of decline, highlighting the limitations of current risk assessment tools in accuracy, complexity, and cost, and the need for improved predictive models. To address this gap, we developed and validated a deep learning model to improve short-term mortality prediction in heart failure patients.

**Methods:**

In this retrospective study, we leveraged the Medical Information Mart for Intensive Care IV database to develop HF-ECGNet, combining an EfficientNet neural network and a Transformer architecture. We also developed a composite model integrating electrocardiogram-based (ECG) predictions and clinical features. We evaluated model performance using the area under the curve (AUC) and other metrics, with gradient-weighted class activation mapping (Grad-CAM) and Shapley additive explanations (SHAP) analyses for interpretability. We conducted comparisons with N-terminal pro-B-type natriuretic peptide and sequential organ failure assessment (SOFA) scores.

**Results:**

We analysed a total of 104 844 ECGs from 36 222 admissions. HF-ECGNet achieved an AUC of 0.664 for the first ECG during initial admission, improving to 0.721 for the last ECG. Incorporating three-day ECG data further enhanced performance, with AUCs of 0.691 (first admission) and 0.698 (last admission). HF-ECGNet outperformed NT-proBNP and SOFA. A composite model integrating ECG data and clinical features achieved the highest AUC of 0.725. Grad-CAM identified critical ECG patterns, while SHAP analysis highlighted ECG-derived features as the most influential predictors.

**Conclusions:**

HF-ECGNet demonstrates potential as a powerful tool for predicting short-term mortality in heart failure patients. Its innovative architecture and integration of clinical data enable more accurate and interpretable risk stratification. Future multi-centre validation is the critical step to fully ascertain its clinical utility and generalisability.

Heart failure is a chronic condition that affects approximately 64.3 million people worldwide [1]. In the USA, the mortality rate of cardiovascular diseases, particularly heart failure, has markedly increased following a long period of decline and stabilisation, with this trend becoming especially pronounced after the COVID-19 pandemic [2]. In Europe, heart failure patients face a poor prognosis, with in-hospital mortality rates between 2% and 17% and an average lifespan reduction of 1.1 to 2.3 years [3–5]. This trend is particularly alarming for hospitalised patients, as up to one in six individuals succumb either during their hospital stay or within 30 days [6]. Therefore, early and precise prediction of mortality risk in this patient population is of key importance. However, numerous existing risk assessment instruments, including the EAHFE-3D scale, the Sequential Organ Failure Assessment (SOFA) score, and various biomarkers such as N-terminal pro-B-type natriuretic peptide (NT-proBNP), troponin, and mid-regional pro-adrenomedullin, have shown to be inadequate for predicting short-term mortality in clinical environments. This inadequacy is attributed to their limited predictive accuracy, complexity, and high costs [7–12]. For example, although the SOFA score offers some predictive utility, it frequently requires extensive data collection and is plagued by calibration issues [8,13]. Similarly, NT-proBNP is useful for diagnosing heart failure but may not reliably predict individual outcomes [14,15]. These limitations underscore the need for simpler and more cost-effective mortality risk assessment tools.

An electrocardiogram (ECG) can detect various arrhythmias and other cardiac abnormalities related to the onset and progression of heart failure [16,17]. However, complex time-series data from a 12-lead ECG, including numerous interrelated physiological parameters, pose significant challenges for traditional statistical and machine learning approaches. Deep learning algorithms are well-suited for addressing these challenges because they efficiently extract features from complex, high-dimensional ECG signals and identify anomalies. Prior research has successfully applied such an approach to predict various cardiovascular disease outcomes, including disease onset, progression, rehospitalisation, and mortality [18–21].

Building on these findings, we propose applying deep learning to the early 12-lead ECG data from heart failure patients to develop a model that effectively predicts short-term outcomes, including 30-day and in-hospital mortality, while providing interpretable results. This approach may help create tailored treatment plans for high-risk patients, potentially improving survival rates. To verify this hypothesis, we utilise ECG data from hospitalised heart failure patients recorded in the Medical Information Mart for Intensive Care IV (MIMIC-IV) database, which contains comprehensive clinical data, to train and evaluate a deep learning model for predicting short-term outcomes.

## METHODS

We adhered to the JoGH’s Guidelines for Reporting Analyses of Big Data Repositories Open to Public (GRABDROP) for reporting our findings [22].

### Study participants

**Figure 1:**
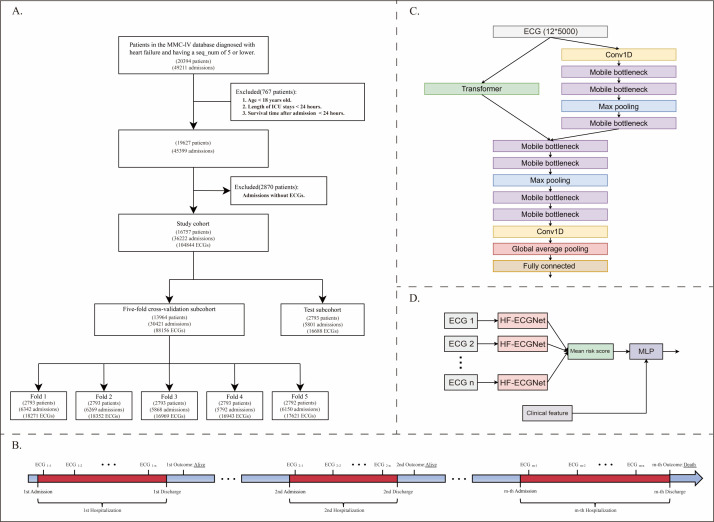
Study outline. **Panel A.** Selected ECGs obtained during hospitalisation matched with short-term outcomes. **Panel B.** Participant selection flowchart. **Panel C.** A novel, lightweight model architecture trained to discriminate short-term mortality outcomes using input from the 12-lead ECG. **Panel D.** A composite model integrated the patient's ECGs from the initial three days along with clinical characteristics. ECG – electrocardiogram, MIMIC-IV – Medical Information Mart for Intensive Care IV, MLP – multilayer perceptron.

Here, we utilised the MIMIC-IV database, version 2.2 (Laboratory for Computational Physiology, Massachusetts Institute of Technology, Cambridge, MA, USA), which encompasses admissions from a hospital in the USA between 2008 and 2019, providing data on approximately 300 000 unique patients across 430 000 hospitalisations [23]. Additionally, we incorporated the MIMIC-IV-ECG, version 1.0 (Laboratory for Computational Physiology, Massachusetts Institute of Technology, Cambridge, MA, USA) module, which specifically focuses on ECG data [24]. The Institutional Review Board at Beth Israel Deaconess Medical Center waived informed consent and permitted the sharing of research resources because all the data were deidentified [25]. The primary investigator (YL) completed all necessary protocols for database access prior to data extraction (record ID 55943213). Here, we focused on adult patients diagnosed with heart failure, as defined by the International Classification of Diseases, 9th and 10th editions (ICD-9 and ICD-10) codes. We included only patients whose sequence number (seq_num), *i.e. *the priority assigned to diagnoses, for heart failure diagnoses ranked within the top five to exclude patients not admitted for heart failure. We excluded patients under 18 years old, those with intensive care unit (ICU) stays shorter than 24 hours, and individuals with brief post-admission survival times to ensure meaningful outcome assessments. The inclusion criterion was at least one 12-lead ECG during hospitalisation with complete waveform images available. We excluded ECGs with missing leads or those containing no values from the analysis. We enrolled patients using the eligibility criteria outlined in the study flowchart and employed a strict patient-level split strategy during data partitioning to ensure data set independence and prevent data leakage (**Figure 1**, Panel A). Specifically, during the initial 5:1 split that created the cross-validation and hold-out test sets, and during the subsequent creation of the 5-folds for cross-validation, we assigned all ECG records exclusively from any individual patient to one subset. We used 5-fold cross-validation to train and validate the model on the cross-validation set. The reporting of this prediction model study adheres to the TRIPOD guideline (Checklist S1 in the **Online Supplementary Document**).

### Clinical and outcome assessments

For each admission, we gathered detailed baseline information, including demographic factors such as age, sex, race, and marital status, as well as comorbid conditions including diabetes, hypertension, chronic kidney disease, acute myocardial infarction (AMI), and chronic obstructive pulmonary disease, which are readily available and have been shown to impact the prognosis of heart failure patients [26–38]. We also collected NT-proBNP and SOFA scores for comparison with the predictive performance of ECG, as they represent clinically validated prognostic indicators in heart failure [8,39,40]. NT-proBNP is a gold-standard cardiac stress biomarker that reflects ventricular wall tension and myocardial dysfunction, with elevated levels strongly correlating with heart failure severity and mortality risk [41]. The SOFA score provides critical prognostic value in acute decompensated heart failure by systematically quantifying multi-organ dysfunction [8]. We included different types of heart failure, such as systolic heart failure, diastolic heart failure, combined systolic and diastolic heart failure, hypertensive heart failure, and other forms of heart failure, categorised by ICD diagnostic codes [42]. The primary outcome was death during hospitalisation or death within 30 days after hospital admission. For patients with multiple heart failure-related admissions, we collected all available ECG data and clinical information.

### ECG assessments

Patient may have had multiple hospital admissions, with at least one ECG recorded per admission (**Figure 1**, Panel B). To maximise the sample size, similar to previous studies, we utilised all the ECG data recorded during hospital stays in the training stage [43,44]. To address class imbalance due to the small number of deceased patients, we randomly matched patients with negative outcomes to those with positive outcomes at a ratio of 2:1 to train the model. The selection of the optimal model is based on the first ECG recorded during the initial admission of the validation set, ensuring the independence of observations in the evaluation of performance metrics. To mitigate bias from transient ECG abnormalities, all ECGs collected within the first three days of admission were repeatedly input into the model, generating individual prediction probabilities that were averaged to obtain the final prediction probability. We then integrated this final value with baseline characteristics to create a composite prediction model *via* a multilayer perceptron, thereby enhancing the model's accuracy and robustness.

### Deep-learning algorithm development

In this study, we utilised a multichannel one-dimensional convolutional EfficientNet as the base architecture because of its established efficiency in feature extraction from ECG signals in prior research [44]. Additionally, we incorporated the Transformer as part of the model architecture to extract shallow features, leveraging its strength in capturing complex temporal relationships within sequential data [45,46]. The combined model was named HF-ECGNet (**Figure 1**, Panel C).

We also built a composite model using a multilayer perceptron with three fully connected layers, which integrated nine indicators: demographics, comorbid conditions, and the three-day average ECG output from HF-ECGNet (**Figure 1**, Panel D).

### Evaluation and comparison

To evaluate individual ECGs *via* HF-ECGNet, we employed gradient-weighted class activation mapping (Grad-CAM) to identify the specific segments of the ECG waveform that contributed significantly to the model's predictions. Furthermore, we compared the predictive performance of HF-ECGNet with that of traditional predictive indicators, including the NT-proBNP and SOFA scores. We also applied SHapley Additive exPlanations (SHAPs) to interpret and visualise the composite model. Lastly, we conducted sensitivity analyses to further evaluate the performance of the HF-ECGNet, whereby we assessed the model at three different time points: the last ECG from the first admission, the first ECG from the final admission, and the last ECG from the final admission.

### Statistical analysis

We expressed categorical variables as frequencies and percentages, and presented continuous variables as the means and standard deviations or as medians (MD) with interquartile ranges (IQRs). We assessed the model performance using the area under the curve (AUC), calibration curve, accuracy, sensitivity, specificity, positive and negative predictive values, F1 score, and Brier score (Text S1 in the **Online Supplementary Document**).

## RESULTS

### Data set and patient demographics

We analysed 36 222 admission records from the MIMIC-IV database, covering the period from 2008 to 2019, which included 104 844 complete 10-second, 12-lead ECGs. We collected these ECGs from 16 757 heart failure patients aged 18 years and older. The distribution of key variables was comparable across the cross-validation set (n = 13 964) and the test set (n = 2793) (**Table 1**). The median age in the cross-validation set was 76 years (IQR = 66–85), and 7375 patients (52.8%) were male. In terms of racial distribution, White individuals constituted the largest group with 10 214 people (73.1%), followed by Black (12.1%), Hispanic (3.4%), or Asian (2.1%) individuals, and people of other races (9.2%). We recorded the average values of the NT-proBNP and SOFA scores during the first three days post-admission. Owing to substantial missing data for these indicators, we provided statistical descriptions of the available data. For NT-proBNP, data were available for 1712 patients in the cross-validation set, with a median of 3321 pg/mL, and for 349 patients in the test set, with a median of 3512 pg/mL. For the SOFA score, data were available for 4306 patients in the cross-validation set, with a median of 3.11 (IQR = 1.77–4.92), and for 888 patients in the test set, with a median of 3.00 (IQR = 1.74–4.94). We recorded short-term mortality rate in 6.5% of patients (912 cases in the cross-validation set and 182 in the test set) (Table S2 in the **Online Supplementary Document**).

**Table 1:** Patient characteristics at first heart failure admission

	CV subcohort (n = 13 964), n (%)	Test subcohort (n = 2793), n (%)
**Age in years**		
<60	1950 (14)	391 (14)
60–70	2680 (19.2)	499 (17.9)
70–80	3509 (25.1)	728 (26.1)
80–90	4003 (28.7)	809 (29)
≥90	1822 (13)	366 (13.1)
**Sex**		
Female	6589 (47.2)	1329 (47.6)
Male	7375 (52.8)	1464 (52.4)
**Race**		
White	10214 (73.1)	2043 (73.1)
Black	1695 (12.1)	319 (11.4)
Hispanic	481 (3.4)	85 (3)
Asian	287 (2.1)	55 (2)
Other	1287 (9.2)	291 (10.4)
**Marital status**		
Married	6167 (44.2)	1281 (45.9)
Divorced	1050 (7.5)	191 (6.8)
Single	3059 (21.9)	592 (21.2)
Widowed	3198 (22.9)	626 (22.4)
Other	490 (3.5)	103 (3.7)
**Diabetes**		
Yes	5727 (41)	1071 (38.3)
No	8237 (59)	1722 (61.7)
**Hypertension**		
Yes	3722 (26.7)	724 (25.9)
No	10242 (73.3)	2069 (74.1)
**CKD**		
Yes	1372 (9.8)	272 (9.7)
No	12592 (90.2)	2521 (90.3)
**AMI**		
Yes	1980 (14.2)	401 (14.4)
No	11984 (85.8)	2392 (85.6)
**COPD**		
Yes	797 (5.7)	158 (5.7)
No	13167 (94.3)	2635 (94.3)
NT-proBNP (pg/mL)*	3321.00 (1370.50–9070.50)	3512.00 (1259.00–8516.00)
SOFA score*	3.11 (1.77–4.92)	3.00 (1.74–4.94)
**Short-term mortality**		
Yes	912 (6.5)	182 (6.5)
No	13 052 (93.5)	2611 (93.5)

CV – cross-validation, CKD – chronic kidney disease, AMI – acute myocardial infarction, COPD – chronic obstructive pulmonary disease, NT-proBNP – N-terminal pro-B-type Natriuretic Peptide, SOFA – sequential organ failure assessment

*Values presented as median (interquartile range). Sample sizes are 1712 (CV subcohort) and 349 (test subcohort) for NT-proBNP, and 4306 (CV subcohort) and 888 (test subcohort) for SOFA score.

### Model performance

We optimised the performance of the HF-ECGNet algorithm *via* 5-fold cross-validation. In the test data set, the algorithm achieved an AUC of 0.664 (95% confidence interval (CI) = 0.658–0.669). The model demonstrated lower performance on the first ECG compared to the other three time points. Specifically, the AUC at the last ECG of the first admission was 0.721 (95% CI = 0.710–0.732), that for the first ECG at the last admission was 0.675 (95% CI = 0.670–0.679), and that for the last ECG at the last admission was 0.711 (95% CI = 0.704–0.717). The predictive accuracy of the algorithm improves as the ECG is taken closer to the outcome (Figure S1 in the **Online Supplementary Document**).

We also provided three Grad-CAM-derived class activation maps from the test set, all representing correctly classified positive ECG results (Figure S2 in the **Online Supplementary Document**). The heatmaps highlight the waveform segments that most strongly activated the final convolutional layer, corresponding to the most significant portions of the image. In Panel A, the model’s strongest activations occurred over multiple consecutive cardiac cycles characteristic of sinus tachycardia. In Panel B, high activation was localised to irregular-rhythm segments in an ECG with sinus arrhythmia. In Panel C, the model focused on premature ventricular contractions. This congruence between the model's attention and known high-risk patterns provides preliminary validation that the model has learned to recognise clinically relevant features from raw data.

### Comparison of HF-ECGNet with traditional metrics

We used HF-ECGNet to process the ECG waveforms from the three days, and we calculated the average output. This approach improved the AUC to 0.691 (95% CI = 0.685–0.698) for the first admission and 0.698 (95% CI = 0.692–0.705) for the last admission (**Table 2**). We compared the average predicted risk from the three days of ECGs with the NT-proBNP and SOFA scores. In the test set, traditional NT-proBNP achieved an AUC of 0.598 for distinguishing short-term mortality in heart failure patients. Among the top 40% of patients with NT-proBNP values of 5180 or higher, the odds ratio (OR) for short-term mortality was 1.750. In contrast, when we used HF-ECGNet with three-day ECG data, the AUC reached 0.670 (95% CI = 0.651–0.690), with an OR of 2.730 (95% CI = 2.292–3.168) for the top 40% of patients compared to others. For the SOFA score, when we used a threshold of 2, patients with higher scores had an OR of 3.132 compared to those with lower scores, while a threshold of 5 yielded an OR of 2.318. Applying the SOFA threshold of 2 and 5 to identify the same top 70% and 23% of patients with three-day ECG data yielded ORs of 3.539 (95% CI = 2.866–4.212) and 3.204 (95% CI = 2.667–3.740). The AUC of the ECG data was 0.686 (95% CI = 0.671–0.701), which was greater than the AUC of 0.650 for the SOFA score (Tables S3 and S4 in the **Online Supplementary Document**).

**Table 2:** Discrimination of short-term mortality by the three-day average ECG*

Admission time	Data set	AUC	Accuracy	Sensitivity	Specificity	PPV	NPV	F1 score	Brier score
First admission	Training set	0.812 (0.777–0.846)	0.758 (0.728–0.787)	0.724 (0.692–0.756)	0.760 (0.731–0.789)	0.175 (0.150–0.199)	0.975 (0.972–0.979)	0.281 (0.247–0.315)	0.142 (0.121–0.163)
First admission	Validation set	0.728 (0.704–0.753)	0.730 (0.711–0.749)	0.577 (0.503–0.651)	0.740 (0.719–0.762)	0.134 (0.114–0.153)	0.962 (0.957–0.967)	0.217 (0.186–0.247)	0.148 (0.129–0.168)
First admission	Test set	0.691 (0.685–0.698)	0.728 (0.707–0.749)	0.524 (0.490–0.557)	0.742 (0.718–0.767)	0.125 (0.120–0.129)	0.957 (0.955–0.959)	0.201 (0.196–0.206)	0.150 (0.131–0.168)
Last admission	Training set	0.805 (0.775–0.834)	0.720 (0.692–0.748)	0.754 (0.729–0.778)	0.715 (0.687–0.744)	0.281 (0.255–0.308)	0.952 (0.945–0.958)	0.410 (0.378–0.441)	0.159 (0.141–0.176)
Last admission	Validation set	0.721 (0.714–0.728)	0.693 (0.677–0.708)	0.607 (0.571–0.642)	0.705 (0.684–0.727)	0.232 (0.219–0.246)	0.925 (0.920–0.929)	0.336 (0.321–0.351)	0.167 (0.152–0.182)
Last admission	Test set	0.698 (0.692–0.705)	0.685 (0.668–0.702)	0.576 (0.536–0.616)	0.701 (0.677–0.726)	0.220 (0.215–0.225)	0.919 (0.914–0.924)	0.318 (0.311–0.326)	0.169 (0.154–0.184)

AUC – area under the curve, NPV – negative predictive value, PPV – positive predictive value

*All values are presented alongside 95% confidence intervals.

### Composite model performance

**Figure 2:**
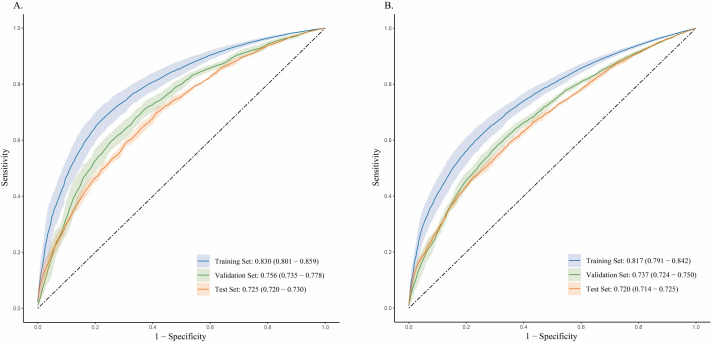
ROC curves for the composite model. **Panel A.** First admission. **Panel B.** Last admission. ROC – receiver operating characteristic.

The composite model that integrates the three-day ECG average from the HF-ECGNet with clinical features achieved an AUC of 0.725 (95% CI = 0.720–0.730) in the test set for the first admission, and 0.720 (95% CI = 0.714–0.725) for the last admission (**Figure 2**). Additionally, this model demonstrated enhanced accuracy, with AUC values of 0.702 (95% CI = 0.683–0.722) for the first admission and 0.667 (95% CI = 0.652–0.682) for the last admission (**Table 3**). The calibration curve revealed strong agreement between predicted probabilities and observed outcomes (Figure S5 in the **Online Supplementary Document**).

**Table 3:** Discrimination of short-term mortality by the three-day average ECG and clinical characteristics*

Admission time	Data set	AUC	Accuracy	Sensitivity	Specificity	PPV	NPV	F1 score	Brier score
First admission	Training set	0.830 (0.801–0.859)	0.730 (0.710–0.751)	0.786 (0.746–0.826)	0.726 (0.706–0.747)	0.166 (0.153–0.180)	0.980 (0.976–0.984)	0.275 (0.254–0.295)	0.153 (0.131–0.175)
First admission	Validation set	0.756 (0.735–0.778)	0.705 (0.685–0.724)	0.680 (0.648–0.713)	0.706 (0.686–0.727)	0.139 (0.123–0.155)	0.970 (0.968–0.972)	0.230 (0.207–0.253)	0.160 (0.137–0.182)
First admission	Test set	0.725 (0.720–0.730)	0.702 (0.683–0.722)	0.616 (0.597–0.634)	0.708 (0.686–0.730)	0.129 (0.123–0.135)	0.963 (0.962–0.965)	0.213 (0.205–0.221)	0.161 (0.140–0.182)
Last admission	Training set	0.817 (0.791–0.842)	0.704 (0.683–0.724)	0.797 (0.761–0.833)	0.690 (0.668–0.712)	0.275 (0.258–0.292)	0.959 (0.951–0.966)	0.409 (0.386–0.431)	0.167 (0.148–0.186)
Last admission	Validation set	0.737 (0.724–0.750)	0.677 (0.661–0.692)	0.685 (0.663–0.708)	0.675 (0.657–0.693)	0.237 (0.221–0.253)	0.936 (0.932–0.940)	0.352 (0.334–0.370)	0.175 (0.157–0.193)
Last admission	Test set	0.720 (0.714–0.725)	0.667 (0.652–0.682)	0.646 (0.611–0.680)	0.671 (0.648–0.693)	0.223 (0.219–0.227)	0.928 (0.924–0.933)	0.331 (0.326–0.336)	0.177 (0.159–0.195)

AUC – area under the curve, NPV – negative predictive value, PPV – positive predictive value

*All values are presented alongside 95% confidence intervals.

### Feature importance and SHAP analysis

**Figure 3:**
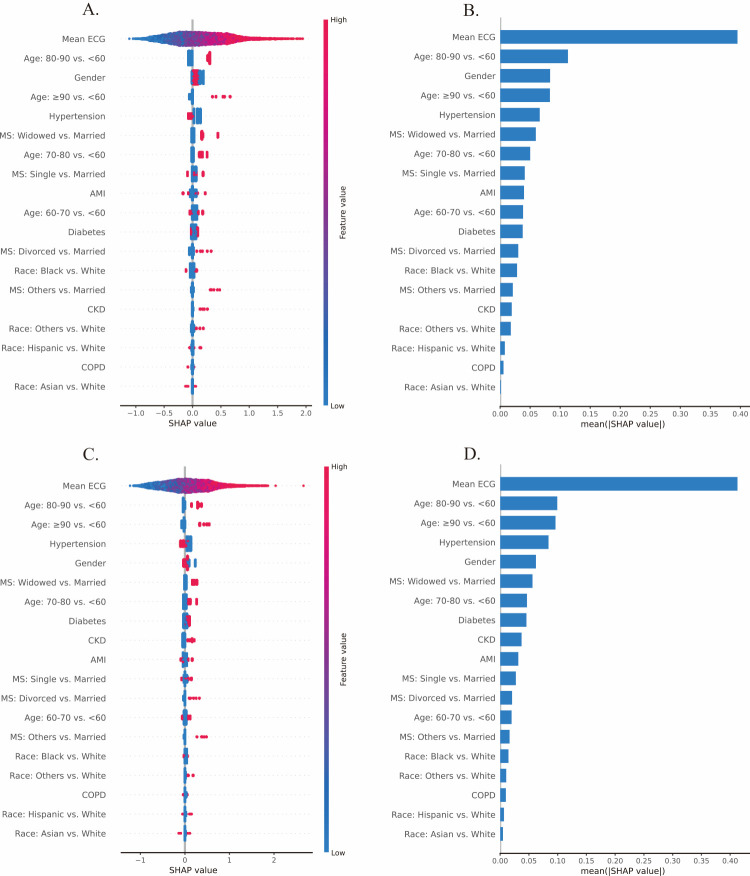
SHAPs analysis illustrating the importance of features and their contributions to the model's predictions. **Panel A.** and **Panel C.** Overall feature importance. **Panel B.** and **Panel D.** Bar charts of the feature contributions. The upper plots correspond to the first admission, and the lower plots relate to the last admission. SHAPs – SHapley Additive exPlanations.

The synthesis of five sets from the 5-fold cross-validation is presented (**Figure 3**). We identified the average risk score derived from the initial three days of ECGs as the most crucial feature for predicting short-term mortality risk in heart failure patients, exerting a prominent positive impact on the model's output, with an average influence magnitude of 34.73%. Age also emerged as an important predictor, with older patients facing a higher risk of mortality. The inclusion of age provided complementary insights that enhanced the predictive accuracy of HF-ECGNet, thereby improving the overall performance of the composite model (Text S2 in the **Online Supplementary Document**).

## DISCUSSION

In this study, we developed and evaluated the HF-ECGNet, which shows considerable promise in predicting short-term mortality among heart failure patients. Our findings indicate that the HF-ECGNet outperforms NT-proBNP and SOFA scores in this cohort, achieving higher accuracy and AUC, particularly when analysing ECG data collected within the first three days of hospital admission. This capability enhances early risk assessment. Moreover, integrating HF-ECGNet with clinical features markedly increases its predictive power, allowing clinicians to accurately identify patients at elevated risk of short-term mortality.

Previous studies have utilised ECG parameters, such as P wave duration, QRS duration, and QT interval to predict cardiovascular mortality [17,47]. However, these tools often fail to fully capture the complexity and subtle variations of ECG waveforms, limiting their effectiveness in clinical applications [48,49]. Additionally, relying solely on ECG reports without analysing waveform information may not provide sufficient clinical insights, potentially impacting the accurate diagnosis and risk assessment of heart diseases [50]. In contrast, HF-ECGNet automatically learns discriminative signals directly from the ECG waveforms, as the model attends to established clinical features such as sinus tachycardia and arrhythmia, demonstrating that it has learned to focus on clinically relevant patterns. However, it also means the model operates as a ‛black box’ whose internal reasoning is not directly accessible. The results from Grad-CAM in this study, which focused on correct positive predictions, primarily highlighted known abnormalities. While this serves to validate the model's clinical relevance, future work is needed to decipher the more complex and potentially novel features the model may use.

Our findings reveal that the predictive performance of ECG data improves as the recording time approaches the outcome event. This is consistent with clinical intuition, as a patient’s deteriorating condition is often reflected in increasingly pronounced ECG abnormalities. HF-ECGNet integrates EfficientNet and Transformer to jointly exploit their complementary strengths: EfficientNet efficiently extracts local morphological features (*e.g.* ST-segment changes or arrhythmic patterns) through depth-wise convolutions and compound scaling, while the Transformer architecture captures long-range temporal dependencies across the ECG recording *via* self-attention [51–53]. This combination enables simultaneous modelling of both localised abnormalities and global dynamic trends that precede adverse events. By fusing convolutional and attention-based mechanisms, HF-ECGNet overcomes the limitations of each architecture used alone, achieving a favourable balance between computational efficiency, data efficiency, and predictive performance, which is essential for practical clinical deployment. This hybrid approach enables HF-ECGNet to process high-dimensional ECG inputs more effectively, extracting nuanced patterns crucial for precise risk prediction. In addition to its architectural strengths, we validated the model using ECG data from the first and last instances, as well as the first three days, during patients' initial and final hospital admissions. These validations highlighted the sensitivity of HF-ECGNet to ECG data at various time points.

The lower model AUC observed in female patients over 80 may be attributable to the pathophysiology of heart failure in this demographic, which is frequently heart failure with preserved ejection fraction, a condition with often subtle and less stereotypical electrocardiographic manifestations [54,55]. In contrast, the model demonstrated enhanced predictive ability in patients with comorbidities such as diabetes, hypertension, or AMI. It is plausible that these conditions promote discernible structural remodelling, including myocardial fibrosis and left ventricular hypertrophy, thereby generating a more distinct electrophysiological substrate that facilitates model interpretation [56-58]. Collectively, these observations emphasise that the reliability of HF-ECGNet is context-dependent and must be interpreted in light of individual patient profiles. Moreover, we developed the model primarily on older, White ICU patients from a single USA academic centre, limiting its generalisability. Although we detected no significant performance gaps across race or heart failure subtypes in this study, the underrepresentation of non-White individuals limits confidence in equitable performance, highlighting the need for validation in diverse, real-world cohorts prior to clinical use.

Low positive predictive value is an unavoidable constraint in predicting rare outcomes, especially in settings where event rates are low. Although high sensitivity supports early detection, it results in a large number of patients being classified as ‛high-risk’ despite a low likelihood of death. In resource-constrained health systems, such false-positive alerts can divert scarce clinical attention, contribute to alert fatigue, and ultimately undermine trust in digital tools. Conversely, a higher decision threshold yields fewer alerts but identifies a smaller group with substantially elevated risk. Given that ECG is widely available, non-invasive, and incurs minimal marginal cost, our model is best deployed not as a universal screening tool, but as a targeted risk-stratification aid, helping clinicians prioritise high-intensity interventions for those most likely to benefit. This approach may support more efficient use of limited clinical resources by helping to direct intensive interventions toward patients at substantially higher risk.

While the HF-ECGNet achieves promising results, this study has several limitations that warrant attention. First, the most significant limitation is the lack of external validation. We developed and validated the model using data sourced solely from a single centre. This inherently limits the assessment of the model's robustness and generalisability, and its applicability to other geographic regions or healthcare systems remains unproven. Additionally, the data set's scope may not fully capture the broader population of heart failure patients. This limitation could affect the model's applicability across diverse patient groups and healthcare settings. Moreover, relying on a single ECG for analysis may not provide enough information, potentially overlooking critical warning signs. Although we used the NT-proBNP and SOFA scores for comparison, they may not serve as ideal benchmarks since they are the only available data in the current database. We recognise that the current model performance, while constituting a step forward, remains below the threshold for definitive clinical decision-making. This underscores the need for further optimisation, including the integration of longitudinal ECG monitoring and multimodal clinical data, to improve predictive power. These limitations suggest that, despite its potential, HF-ECGNet requires external validation and further refinement to ensure its efficacy across various clinical environments. Addressing these issues in future research will be crucial for translating the model's capabilities into practical and widespread clinical applications.

## CONCLUSIONS

HF-ECGNet represents a meaningful advancement in predicting short-term mortality among patients with heart failure. By integrating the EfficientNet architecture, which excels at capturing local morphological features, with the Transformer block, which effectively models global contextual relationships, our framework enables a comprehensive analysis of ECG signals. This hybrid approach, further enhanced by the incorporation of clinical features, significantly improves predictive performance. As a proof of concept, HF-ECGNet demonstrates that ECG-only models can approach or even slightly surpass traditional scores, thereby establishing a valuable theoretical and methodological foundation for future research. However, before any claim of practice-changing impact can be made, prospective external validation and integration with richer longitudinal data are warranted. If such efforts prove successful, this approach could provide meaningful support for heart failure management, particularly in resource-limited settings where efficient and accessible diagnostic tools are urgently needed.

## Additional material


Online Supplementary Document
