# Supplementary material for: Development and explanation of electrocardiogram-based deep learning for predicting short-term mortality in heart failure patients
Source: J Glob Health. 2026 Feb 13;16:04048. doi: 10.7189/jogh.16.04048 (PMC12900550; doi:10.7189/jogh.16.04048)
Supplement: Online Supplementary Document [file jogh-16-04048-s001.pdf]

Supplement to: Li Y, Cheng L, Zhou H, Yu H, Liu Z, Zhang Q. Development and explanation of electrocardiogram-based deep learning for predicting short-term mortality in heart failure patients. J Glob Health. 2026;16:04048.

Development and Explanation of Electrocardiogram-Based Deep Learning for Predicting Short-Term Mortality in Heart Failure Patients

Text S1:

Methods

We adhered to the JoGH’s Guidelines for Reporting Analyses of Big Data Repositories Open to Public (GRABDROP) for reporting our findings [1].

ECG assessments

ECG (Electrocardiogram) waveform data were collected at 500 Hz and extracted as 12 × 5000 amplitude matrices, representing complete 10-second continuous recordings for all 12 leads. A median filter was used to eliminate baseline drift from the raw waveforms.

Deep-learning algorithm development

HF-ECGNet takes a 12-channel vector of length 5000 as input, with the data being duplicated and simultaneously fed into both the convolutional neural network and the Transformer during the input stage. The features extracted by these two algorithms are then fused at an intermediate stage to capture complex high-dimensional features. The training labels for the model were based on in-hospital mortality or mortality at 30 days. We initialized the model with random weights and trained it via the AdamW optimizer with focal loss as the loss function. The batch size was set to 64, with an initial learning rate of 0.001, and the activation function used was leaky ReLU. Training was stopped early on the basis of the area under the curve (AUC) from the validation dataset.

The folds of the cross-validation data used in the multilayer perceptron (MLP) were consistent with those from the HF-ECGNet. The model was designed with an input length of 10, while the output represented the patient's survival outcome. Similar to the first model, the MLP uses the AdamW optimizer and focal loss as the loss

function, with a batch size of 64. However, the learning rate was increased to 0.01, and the activation function was changed to ReLU. Training was stopped early on the basis of the AUC of the validation set. Both models were trained via the PyTorch library.

Evaluation and comparison

To comprehensively evaluate the performance of HF-ECGNet across diverse clinical phenotypes, we conducted subgroup analyses to identify patient subgroups in which the model demonstrated high AUC as well as those with suboptimal performance. For the gradient-weighted class activation mapping (Grad-CAM), feature maps were extracted from the final convolutional layer of HF-ECGNet, and gradients were computed at this layer to generate class activation maps. These maps facilitated the visualization of key regions influencing the model's decision-making process, thereby enhancing our understanding of its predictive capabilities. For effective visualization, the ECG signals were converted into images using the ecg-plot library, with images stored at a resolution of 100 DPI to ensure clarity. The extracted class activation features were subsequently overlaid onto the ECG images with an alpha transparency of 0.7, providing a clear representation of critical areas while preserving the integrity of the underlying waveform details.

To compare HF-ECGNet with traditional predictive indicators, we included ECG data, N-terminal pro B-type natriuretic peptide (NT-proBNP) levels, and sequential organ failure assessment (SOFA) scores recorded during the first three days after admission and calculated their average values. Given the high missingness of NT-proBNP and SOFA scores, HF-ECGNet was compared with NT-proBNP among patients with ECG and NT-proBNP data, and with SOFA among those with ECG and SOFA data, with performance assessed separately in each subgroup. In each subcohort, entropy balancing was used to ensure covariate balance with the full study population before performance comparison (see Statistical Analysis Section in the Online Supplementary Document). The odds ratios (ORs) of these two predictive factors were compared against the outputs of HF-ECGNet. The assessment of the HF-

ECGNet included NT-proBNP levels exceeding 5180, a threshold recognised as effective for evaluating short-term prognosis in patients with acute heart failure [2]. The ORs, sensitivity, and specificity for mortality among heart failure patients at this NT-proBNP threshold were then estimated. Similarly, we compared HF-ECGNet with SOFA scores by the same methodology, setting the SOFA threshold at 2 or 5[3,4]. To further evaluate clinical utility, we calculated the number needed to predict (NNP) by comparing HF-ECGNet with NT-proBNP and SOFA.

Shapley additive explanation (SHAP) values offer a quantitative measure of each feature's contribution to the final prediction, enabling the assessment of the significance of various features and baseline characteristics in the model's decision-making process. In addition, global SHAP values were visualized to elucidate the impact of different features on the assessment of short-term mortality risk in heart failure patients, highlighting the relative importance of various predictors and enabling clinicians to readily identify critical factors for risk assessment.

To better understand the composite model's performance in key patient populations, we conducted independent analyses focused on distinct heart failure types categorized by international classification of diseases (ICD) diagnostic codes: (1) systolic heart failure, (2) diastolic heart failure, (3) combined systolic and diastolic heart failure, and (4) hypertensive heart failure. By segmenting the patient population on the basis of these classifications, we aimed to assess the model's predictive accuracy and reliability across diverse clinical scenarios. This approach enabled us to determine whether the model maintained consistent performance across different heart failure types, which is essential for its potential implementation in clinical practice.

Sensitivity analyses, which assessed the model at three different time points, ensured the model's generalizability across different time points and admission records, facilitating a comprehensive evaluation of its effectiveness and stability in real-world clinical scenarios. Additionally, we conducted sensitivity analyses on the composite model to account for potential confounding from prolonged hospitalizations by re-evaluating the primary outcome while excluding in-hospital deaths that occurred more than 30 days after admission.

Statistical analysis

To compare HF-ECGNet with NT-proBNP and SOFA, both of which exhibit substantial missingness, we restricted analyses to patients with complete ECG and comparator data, respectively. Because these subsets may differ systematically from the full cohort, we applied entropy balancing within each fold of the cross-validation and in the test set separately to reweight each subset so that its distribution of baseline covariates matched that of the full cohort, using a standardized mean difference (SMD) threshold of $|SMD| < 0.1$ to define adequate balance. All subsequent comparisons involving these two subsets were conducted using the derived entropy-balancing weights.

To correct for potential miscalibration induced by the 2:1 case–control matching during training, predicted probabilities were post-processed using Platt scaling. 95% confidence intervals (CIs) were calculated on the basis of the results from fivefold cross-validation. All model development and statistical analyses were performed via Python, version 3.8.0 or R, version 4.3.1.

Text S2:

Results

Comparison of HF-ECGNet with Traditional Metrics

When comparing HF-ECGNet to NT-proBNP, the NNP was 41.109 in the test set, indicating that for every 41 additional patients identified by HF-ECGNet beyond NT-proBNP, one additional death could be predicted. Similarly, when compared to SOFA scores, the NNP values were lower at 21.320 in the test set, demonstrating greater predictive efficiency over SOFA (Table S5).

Subgroup analyses based on the test set revealed notable variations in model performance across patient strata defined by age, sex, diabetes, hypertension, and acute myocardial infarction (AMI) between the first and last hospital admissions (Figure S3). Further evaluation identified that the model exhibited suboptimal performance among female patients aged over 80 years (Figure S4, Panel A).

Conversely, HF-ECGNet demonstrated higher predictive accuracy in patients with at least one comorbidity, including diabetes, hypertension, or AMI, compared to those without any of these conditions (Figure S4, Panel B).

Composite Model Performance

The performance of composite model for the first admission was further evaluated at different thresholds across the training, validation, and test sets (Table S6). At a threshold of 0.3, the model exhibited high sensitivity (0.881) but low specificity (0.360) on the test set, resulting in a positive predictive value (PPV) of only 0.089. In contrast, when the threshold was increased to 0.6, sensitivity decreased to 0.152, while specificity rose markedly to 0.962, and the PPV improved to 0.246. Furthermore, the sensitivity analysis indicated that the composite model's performance remained stable (AUC of 0.733 in the first admission test set compared to 0.725 in the original analysis), even after excluding patients who died in the hospital more than 30 days after admission (Table S7).

Subtype Analysis

In the Subtype analysis, the combined systolic and diastolic heart failure subtype achieved the highest AUC of 0.794 (95% CI = 0.770-0.818), with a sensitivity of 0.817 (95% CI = 0.756-0.878) and specificity of 0.561 (95% CI = 0.513-0.609). HF-ECGNet also demonstrated robust performance across other heart failure subtypes, with minimal variation in overall predictive efficacy. Furthermore, we analysed ECG data from the first three days following the last admission (Figure S6 and Table S8).

REFERENCES

1. Rudan I, Song P, Adeloye D, Campbell H. Journal of global health's guidelines for reporting analyses of big data repositories open to the public (GRABDROP): Preventing "paper mills", duplicate publications, misuse of statistical inference, and inappropriate use of artificial intelligence. J Glob Health 2025;15:01004.

2. Januzzi JL, Van Kimmenade R, Lainchbury J, Bayes-Genis A, Ordonez-Llanos J, Santalo-Bel M, et al. NT-proBNP testing for diagnosis and short-term prognosis in acute destabilized heart failure: an international pooled analysis of 1256 patients. Eur Heart J 2006;27(3):330–7.

3. Elias A, Agbarieh R, Saliba W, Khoury J, Bahouth F, Nashashibi J, et al. SOFA score and short-term mortality in acute decompensated heart failure. Sci Rep 2020;10(1):20802.

4. Jentzer JC, Bennett C, Wiley BM, Murphree DH, Keegan MT, Gajic O, et al. Predictive value of the sequential organ failure assessment score for mortality in a contemporary cardiac intensive care unit population. J Am Heart Assoc 2018;7(6):e008169.

(The code and models used in this study are available from the corresponding author: zhangqj@hrbmu.edu.cn.)

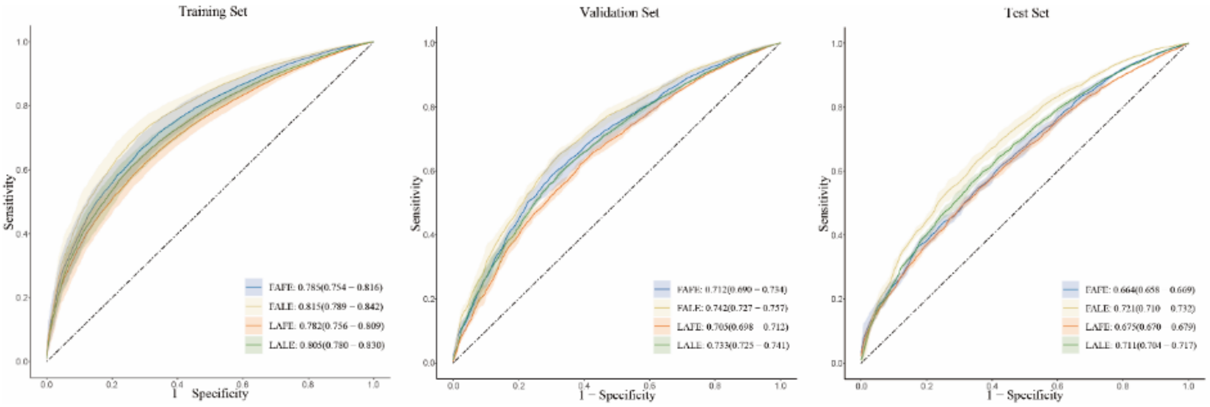

Figure S1. Receiver-operating curves for HF-ECGNet on the training, validation, and test datasets at four different time points.

FAFE – First Admission First ECG, FALE – First Admission Last ECG, LAFE – Last Admission First ECG, LALE – Last Admission Last ECG.

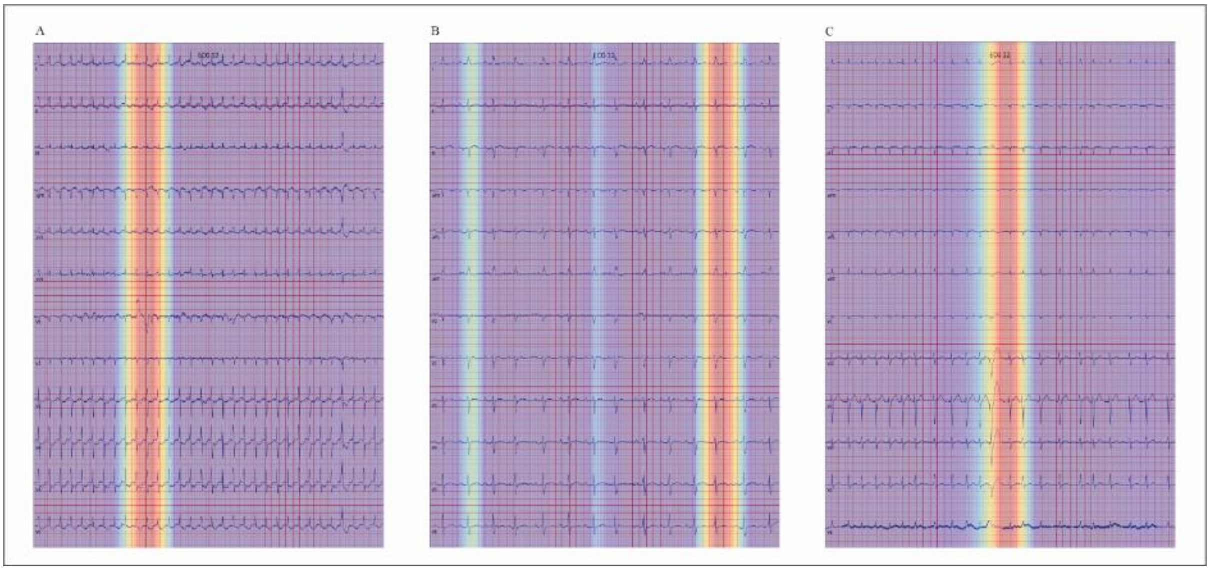

Figure S2. Selected ECGs with positive results during hospitalization and correctly classified, highlighting the most relevant features identified through Class Activation Mapping (CAM) analysis, where the red color typically represents regions of the input that have the highest importance or contribution to the predicted class.

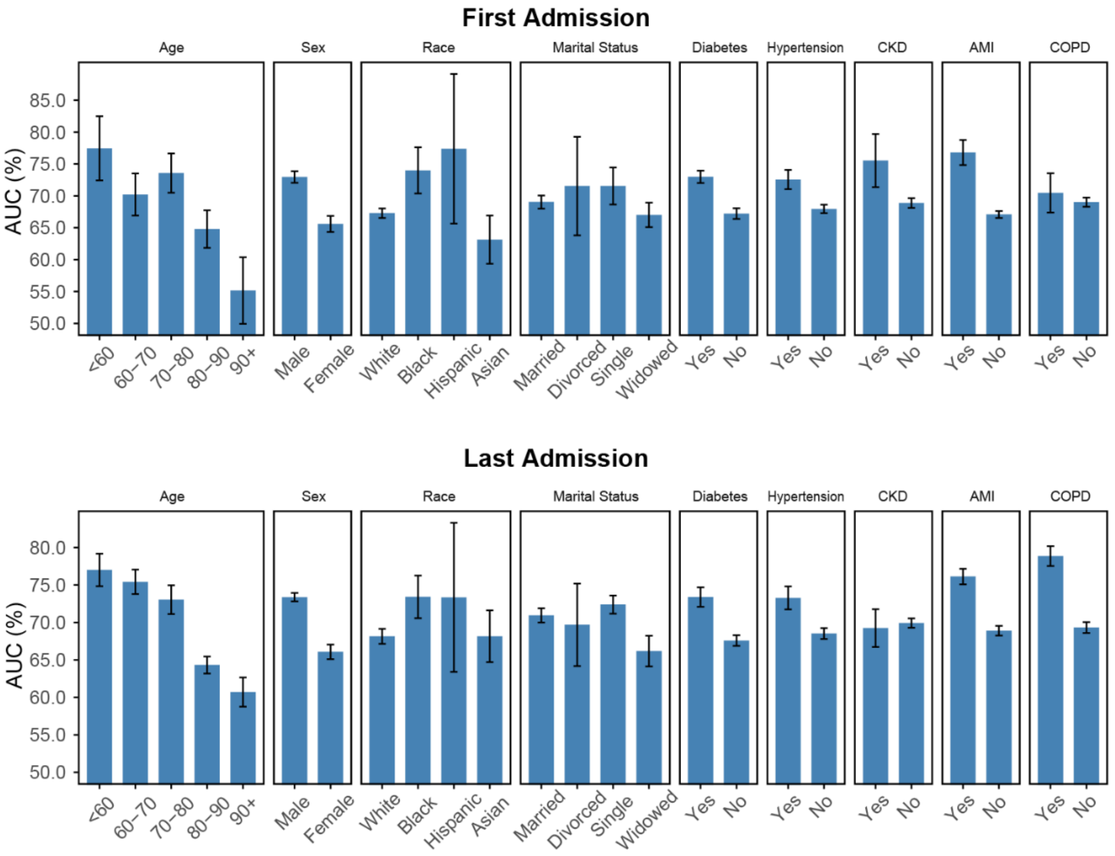

Figure S3. Subgroup analyses stratified by age, sex, race, marital status, diabetes, hypertension, chronic kidney disease, acute myocardial infarction and chronic obstructive pulmonary disease.

AUC – Area Under the Curve.

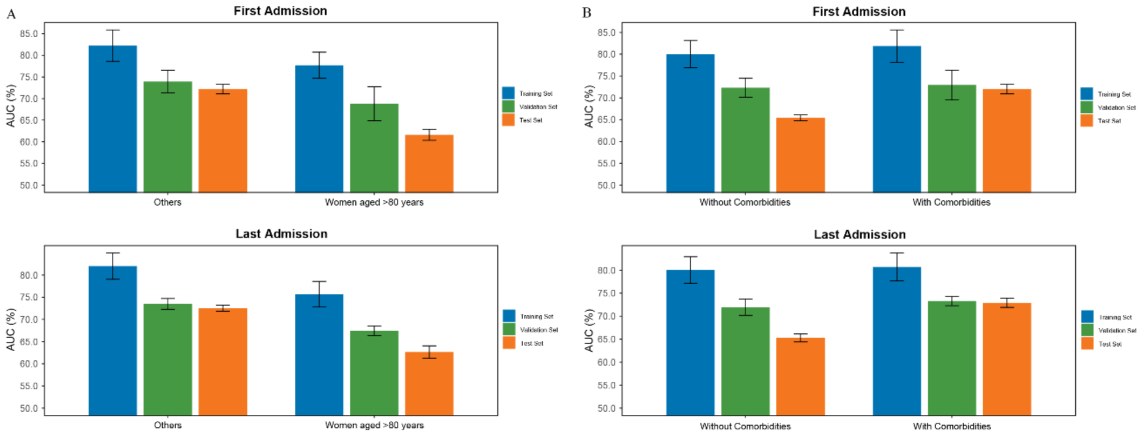

Figure S4. Model performance in stratified patient. **Panel A.** Area under the curve (AUC) performance of HF-ECGNet in female patients aged over 80 years compared to all other patients. **Panel B.** Comparative AUC of HF-ECGNet between patients with at least one comorbidity (diabetes, hypertension, or acute myocardial infarction) and those without any of these conditions.

AUC – Area Under the Curve.

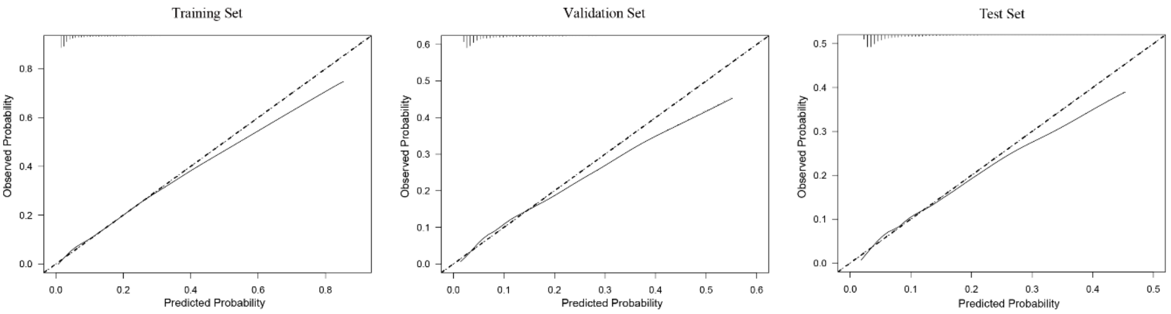

Figure S5. Calibration curve for the composite model constructed by 3-day average ECG and clinical features.

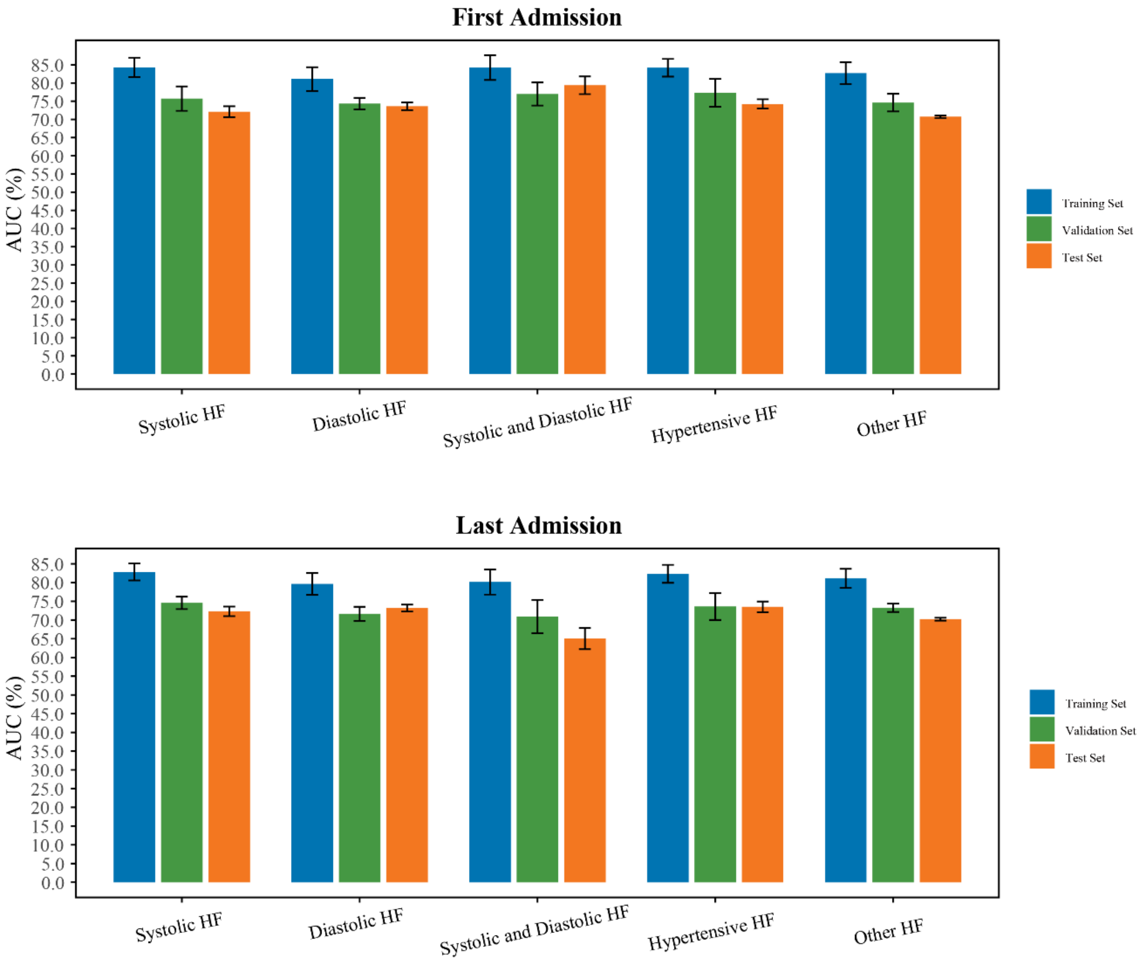

Figure S6. Area under the curve (AUC) values for the training, validation, and test datasets across different subtypes.

HF – heart failure. AUC – Area Under the Curve

Table S1. Outline of JoGH guideline items

JoGH guideline item	Author's Response
1. Please list all papers published by each co-author in previous 3 years that were based on secondary analysis of a big data repository	1. Tian W, Zhang Y, Han X, **Li Y**, Liu J, Wang H, **Zhang Q**, Ma Y, Yan G. Development and validation of a predictive model for depression risk in the U.S. adult population: Evidence from the 2007-2014 NHANES. BMC Psychol. 2023;11:244.<br>2. Mai L, Yang X, Shao C, **Yu H**, Du F, Liu J, Guan Y, Li H, Yang M, Meng H, **Zhang Q**. Enhanced tumor heterogeneity mediates poor prognosis in females with TP53 mutation in lung adenocarcinoma. Int J Biol Macromol. 2025;:149083.<br>3. Liu J, Han X, Chen L, Mai L, Su X, Dong Y, Wang B, **Zhang Q**. The association between diet-exercise patterns and cirrhosis: A cross-sectional study from NHANES 2017-march 2020. Nutrients. 2024;16:1617.<br>4. Yan G, **Zhang Q**, Yan Y, Zhang Y, **Li Y**, Liu M, Tian W. Trends in the prevalence and treatment of comorbid depression among US adults with and without cancer, 2005-2020. J Affect Disord. 2023;340:743 - 750.
2. Please explain the key elements of your study design and the use of the available datasets that make your study an original scientific contribution	To our knowledge, this is the first study to predict short-term mortality in heart failure patients directly from raw ECG signals using a deep learning approach. This study leverages the MIMIC-IV database—including 36,222 hospital admissions and 104,844 ECGs—to develop HF-ECGNet, a novel deep learning model that combines EfficientNet and Transformer architectures for predicting short-term mortality in heart failure patients. HF-ECGNet outperforms conventional clinical tools such as NT-proBNP and SOFA score, with performance further enhanced when integrating dynamic ECG data from the first three hospital days with clinical features. Using interpretable techniques (Grad-CAM and SHAP), the model's focus on clinically relevant ECG segments was validated against established knowledge. By delivering a low-cost, non-invasive, and interpretable risk stratification tool, this work establishes a new paradigm for ECG-based prognostication in heart failure.
3. Please list all publications that addressed similar research questions in the same dataset	Previous studies using the MIMIC-IV database, some cited in the Introduction and in relation to heart failure ICD codes, mainly employed traditional statistical methods and conventional clinical or

and indicate where you cited them in your paper	hematological variables. In contrast, our study applies deep learning to raw ECG signals, addressing a distinct research question. While we acknowledge these prior works for context, they do not overlap substantially with our approach or objectives.
4. Please explain how you addressed multiple testing through an appropriately rigorous statistical threshold and indicate this in the methods section	We addressed multiple testing by pre-specifying short-term mortality as the primary outcome and AUC as the main performance metric, with all analyses (including comparisons to NT-proBNP/SOFA and composite modeling) hypothesis-driven. Model performance was rigorously evaluated using five-fold cross-validation to mitigate overfitting and ensure robustness. This approach, focused on a unified predictive framework rather than multiple independent hypothesis tests, aligns with standard practice in clinical machine learning studies.
5. Please declare to what extent have AI chatbots been used in developing your paper and to which parts of the paper did they contribute	AI chatbots were used minimally in the development of this manuscript, solely for assistance with English vocabulary selection and grammar correction to improve language clarity.

Table S2. Characteristics of the Cross-validation Cohort

	**CV Fold 1** (N=2793)	**CV Fold 2** (N=2793)	**CV Fold 3** (N=2793)	**CV Fold 4** (N=2793)	**CV Fold 5** (N=2792)
**Age**					
<60	395 (14.1%)	426 (15.3%)	389 (13.9%)	378 (13.5%)	362 (13%)
60-70	549 (19.7%)	507 (18.2%)	533 (19.1%)	550 (19.7%)	541 (19.4%)
70-80	694 (24.8%)	696 (24.9%)	708 (25.3%)	701 (25.1%)	710 (25.4%)

80-90	783 (28%)	801 (28.7%)	804 (28.8%)	788 (28.2%)	827 (29.6%)
≥90	372 (13.3%)	363 (13%)	359 (12.9%)	376 (13.5%)	352 (12.6%)
**Sex**					
Female	1294 (46.3%)	1301 (46.6%)	1333 (47.7%)	1327 (47.5%)	1334 (47.8%)
Male	1499 (53.7%)	1492 (53.4%)	1460 (52.3%)	1466 (52.5%)	1458 (52.2%)
**Race**					
White	2055 (73.6%)	2018 (72.3%)	2051 (73.4%)	2026 (72.5%)	2064 (73.9%)
Black	338 (12.1%)	344 (12.3%)	343 (12.3%)	334 (12%)	336 (12%)
Hispanic	95 (3.4%)	102 (3.7%)	92 (3.3%)	94 (3.4%)	98 (3.5%)
Asian	49 (1.8%)	54 (1.9%)	55 (2%)	68 (2.4%)	61 (2.2%)
Other	256 (9.2%)	275 (9.8%)	252 (9%)	271 (9.7%)	233 (8.3%)
**Marital Status**					
Married	1276 (45.7%)	1215 (43.5%)	1250 (44.8%)	1190 (42.6%)	1236 (44.3%)
Divorced	219 (7.8%)	225 (8.1%)	190 (6.8%)	224 (8%)	192 (6.9%)
Single	589 (21.1%)	634 (22.7%)	617 (22.1%)	588 (21.1%)	631 (22.6%)
Widowed	608 (21.8%)	619 (22.2%)	648 (23.2%)	682 (24.4%)	641 (23%)
Other	101 (3.6%)	100 (3.6%)	88 (3.2%)	109 (3.9%)	92 (3.3%)
**Diabetes**					
Yes	1107 (39.6%)	1166 (41.7%)	1144 (41%)	1166 (41.7%)	1144 (41%)

No	1686 (60.4%)	1627 (58.3%)	1649 (59%)	1627 (58.3%)	1648 (59%)
**Hypertension**					
Yes	772 (27.6%)	729 (26.1%)	715 (25.6%)	736 (26.4%)	770 (27.6%)
No	2021 (72.4%)	2064 (73.9%)	2078 (74.4%)	2057 (73.6%)	2022 (72.4%)
**CKD**					
Yes	258 (9.2%)	277 (9.9%)	287 (10.3%)	283 (10.1%)	267 (9.6%)
No	2535 (90.8%)	2516 (90.1%)	2506 (89.7%)	2510 (89.9%)	2525 (90.4%)
**AMI**					
Yes	364 (13%)	412 (14.8%)	379 (13.6%)	422 (15.1%)	403 (14.4%)
No	2429 (87%)	2381 (85.2%)	2414 (86.4%)	2371 (84.9%)	2389 (85.6%)
**COPD**					
Yes	171 (6.1%)	156 (5.6%)	161 (5.8%)	150 (5.4%)	159 (5.7%)
No	2622 (93.9%)	2637 (94.4%)	2632 (94.2%)	2643 (94.6%)	2633 (94.3%)
**NT-proBNP (pg/mL)***	3336.50 (1362.00-8741.00) {N=350}	3564.00 (1424.00-10045.00) {N=318}	2934.00 (1264.00-8967.00) {N=374}	3206.25 (1167.00-8439.00) {N=338}	3656.50 (1682.50-9433.50) {N=332}
**SOFA score***	3.01 (1.62-4.71) {N=849}	3.11 (1.76-5.00) {N=846}	3.26 (1.90-4.94) {N=886}	3.09 (1.81-5.00) {N=870}	3.15 (1.79-4.90) {N=855}
**Short-Term Outcomes**					

Yes	170 (6.1%)	201 (7.2%)	189 (6.8%)	176 (6.3%)	176 (6.3%)
No	2623 (93.9%)	2592 (92.8%)	2604 (93.2%)	2617 (93.7%)	2616 (93.7%)

CV – Cross-Validation, CKD – Chronic Kidney Disease, AMI – Acute Myocardial Infarction, COPD – Chronic Obstructive Pulmonary Disease, NT-proBNP – N-terminal pro B-type Natriuretic Peptide, SOFA – Sequential Organ Failure Assessment.

*The values for NT-proBNP and SOFA score represent the median (interquartile range). Sample sizes are N=350, N=318, N=374, N=338 and N=332 for NT-proBNP, and N=849, N=846, N=886, N=870, and N=855 for SOFA score.

Table S3. Discrimination of short-term mortality by the 3-day average ECG or NT-proBNP

	Dataset	AUC	Accuracy	Sensitivity	Specificity	PPV	NPV
3-day ECG	Training Set	0.840 (0.809 - 0.871)	0.758 (0.724 - 0.793)	0.795 (0.766 - 0.824)	0.755 (0.719 - 0.790)	0.231 (0.200 - 0.261)	0.976 (0.971 - 0.980)
3-day ECG	Validation Set	0.768 (0.731 - 0.806)	0.747 (0.727 - 0.767)	0.688 (0.596 - 0.781)	0.753 (0.731 - 0.775)	0.203 (0.161 - 0.246)	0.964 (0.951 - 0.976)
3-day ECG	Test Set	0.670 (0.651 - 0.690)	0.707 (0.683 - 0.731)	0.492 (0.429 - 0.556)	0.726 (0.696 - 0.756)	0.135 (0.123 - 0.147)	0.943 (0.937 - 0.948)
NT-proBNP	Training Set	0.736 (0.720 - 0.752)	0.646 (0.640 - 0.651)	0.699 (0.678 - 0.720)	0.641 (0.636 - 0.646)	0.150 (0.145 - 0.155)	0.959 (0.955 - 0.963)
NT-proBNP	Validation Set	0.739 (0.683 - 0.796)	0.646 (0.622 - 0.669)	0.704 (0.630 - 0.777)	0.641 (0.620 - 0.662)	0.151 (0.129 - 0.173)	0.959 (0.943 - 0.975)
NT-proBNP	Test Set	0.598 (0.598 - 0.598)	0.608 (0.608 - 0.608)	0.523 (0.523 - 0.523)	0.615 (0.615 - 0.615)	0.105 (0.105 - 0.105)	0.937 (0.937 - 0.937)

AUC – Area Under the Curve, PPV – Positive Predictive Value, NPV – Negative Predictive Value, ECG – Electrocardiogram, NT-proBNP – N-terminal pro B-type Natriuretic Peptide.

Table S4. Discrimination of short-term mortality by the 3-day average ECG or SOFA score

	Dataset	AUC	Accuracy	Sensitivity	Specificity	PPV	NPV
3-day ECG	Training Set	0.837 (0.803 - 0.870)	0.739 (0.705 - 0.774)	0.812 (0.784 - 0.841)	0.731 (0.696 - 0.766)	0.266 (0.234 - 0.297)	0.970 (0.964 - 0.976)
3-day ECG	Validation Set	0.744 (0.726 - 0.762)	0.701 (0.683 - 0.719)	0.640 (0.557 - 0.724)	0.708 (0.681 - 0.735)	0.205 (0.182 - 0.227)	0.944 (0.929 - 0.958)
3-day ECG	Test Set	0.686 (0.671 - 0.701)	0.687 (0.668 - 0.706)	0.561 (0.504 - 0.619)	0.702 (0.675 - 0.728)	0.181 (0.171 - 0.191)	0.932 (0.925 - 0.938)
SOFA score	Training Set	0.642 (0.636 - 0.648)	0.387 (0.384 - 0.389)	0.852 (0.847 - 0.858)	0.332 (0.328 - 0.336)	0.131 (0.128 - 0.134)	0.950 (0.948 - 0.952)
SOFA score	Validation Set	0.641 (0.619 - 0.663)	0.387 (0.378 - 0.396)	0.852 (0.831 - 0.873)	0.332 (0.317 - 0.347)	0.131 (0.117 - 0.144)	0.950 (0.940 - 0.960)
SOFA score	Test Set	0.650 (0.650 - 0.650)	0.388 (0.388 - 0.388)	0.863 (0.863 - 0.863)	0.332 (0.332 - 0.332)	0.132 (0.132 - 0.132)	0.954 (0.954 - 0.954)

AUC – Area Under the Curve, PPV – Positive Predictive Value, NPV – Negative Predictive Value, ECG – Electrocardiogram, SOFA – Sequential Organ Failure Assessment score.

Table S5. NNP for HF-ECGNet Compared to NT-proBNP and SOFA Scores

	Training Set	Validation Set	Test Set
HF-ECGNet vs. NT-proBNP	14.203 (8.939 - 19.467)	27.477 (14.107 - 40.846)	41.109 (21.886 - 60.332)

HF-ECGNet vs. SOFA	7.839 (6.133 - 9.544)	14.028 (11.122 - 16.934)	21.320 (16.461 - 26.178)

NNP – Number Needed to Predict

Table S6. Performance of the Composite Model at Different Thresholds

Threshold	Dataset	Accuracy	Sensitivity	Specificity	PPV	NPV
0.3	Training Set	0.409 (0.301 - 0.518)	0.953 (0.920 - 0.985)	0.372 (0.253 - 0.490)	0.097 (0.083 - 0.112)	0.992 (0.989 - 0.995)
	Validation Set	0.394 (0.286 - 0.501)	0.907 (0.877 - 0.937)	0.358 (0.244 - 0.473)	0.092 (0.072 - 0.112)	0.982 (0.980 - 0.984)
	Test Set	0.394 (0.297 - 0.492)	0.881 (0.815 - 0.947)	0.360 (0.252 - 0.469)	0.089 (0.080 - 0.098)	0.979 (0.974 - 0.984)
0.4	Training Set	0.638 (0.546 - 0.730)	0.841 (0.755 - 0.926)	0.624 (0.520 - 0.728)	0.140 (0.117 - 0.162)	0.984 (0.977 - 0.990)
	Validation Set	0.620 (0.525 - 0.714)	0.759 (0.691 - 0.828)	0.610 (0.504 - 0.716)	0.125 (0.095 - 0.156)	0.974 (0.969 - 0.978)
	Test Set	0.615 (0.524 - 0.705)	0.713 (0.604 - 0.823)	0.608 (0.503 - 0.712)	0.116 (0.103 - 0.129)	0.969 (0.962 - 0.977)
0.5	Training Set	0.809 (0.744 - 0.874)	0.634 (0.480 - 0.788)	0.821 (0.741 - 0.901)	0.210 (0.174 - 0.246)	0.971 (0.961 - 0.981)
	Validation Set	0.785 (0.713 - 0.857)	0.518 (0.397 - 0.640)	0.804 (0.718 - 0.889)	0.166 (0.130 - 0.202)	0.961 (0.953 - 0.968)
	Test Set	0.782 (0.713 - 0.851)	0.469 (0.341 - 0.596)	0.804 (0.721 - 0.887)	0.151 (0.131 - 0.171)	0.957 (0.950 - 0.963)

	Training Set	0.922 (0.912 - 0.932)	0.255 (0.104 - 0.405)	0.968 (0.947 - 0.989)	0.377 (0.335 - 0.419)	0.950 (0.941 - 0.958)
0.6	Validation Set	0.910 (0.893 - 0.927)	0.166 (0.056 - 0.276)	0.961 (0.935 - 0.988)	0.242 (0.196 - 0.288)	0.944 (0.936 - 0.951)
	Test Set	0.909 (0.890 - 0.929)	0.152 (0.065 - 0.238)	0.962 (0.936 - 0.989)	0.246 (0.206 - 0.286)	0.942 (0.938 - 0.946)

PPV – Positive Predictive Value, NPV – Negative Predictive Value

Table S7. Sensitivity Analysis of Short-Term Mortality Using 3-Day ECG Averages and Clinical Characteristics

**Admission Time**	**Dataset**	**AUC**	**Accuracy**	**Sensitivity**	**Specificity**	**PPV**	**NPV**	**F1 score**	**Brier score**
First Admission	Training Set	0.831 (0.802 - 0.860)	0.730 (0.710 - 0.751)	0.789 (0.747 - 0.832)	0.726 (0.706 - 0.747)	0.163 (0.149 - 0.177)	0.981 (0.977 - 0.985)	0.270 (0.249 - 0.292)	0.153 (0.130 - 0.175)
First Admission	Validation Set	0.759 (0.739 - 0.780)	0.705 (0.686 - 0.725)	0.686 (0.652 - 0.720)	0.706 (0.686 - 0.727)	0.137 (0.120 - 0.153)	0.971 (0.969 - 0.973)	0.228 (0.203 - 0.252)	0.159 (0.137 - 0.182)
First Admission	Test Set	0.733 (0.727 - 0.739)	0.703 (0.683 - 0.723)	0.627 (0.608 - 0.647)	0.708 (0.686 - 0.730)	0.126 (0.120 - 0.132)	0.966 (0.965 - 0.967)	0.209 (0.202 - 0.217)	0.160 (0.139 - 0.181)

Last Admission	Training Set	0.818 (0.793 - 0.843)	0.704 (0.683 - 0.724)	0.800 (0.763 - 0.837)	0.690 (0.668 - 0.712)	0.270 (0.253 - 0.287)	0.960 (0.953 - 0.968)	0.403 (0.381 - 0.426)	0.166 (0.147 - 0.186)
Last Admission	Validation Set	0.740 (0.725 - 0.754)	0.677 (0.661 - 0.693)	0.689 (0.666 - 0.712)	0.675 (0.657 - 0.693)	0.233 (0.217 - 0.249)	0.938 (0.935 - 0.942)	0.348 (0.328 - 0.368)	0.175 (0.156 - 0.193)
Last Admission	Test Set	0.723 (0.717 - 0.729)	0.668 (0.653 - 0.683)	0.651 (0.616 - 0.686)	0.671 (0.648 - 0.693)	0.219 (0.215 - 0.223)	0.932 (0.927 - 0.936)	0.327 (0.322 - 0.333)	0.176 (0.158 - 0.194)

AUC – Area Under the Curve, PPV – Positive Predictive Value, NPV – Negative Predictive Value.

Table S8. Discrimination of short-term mortality by the average ECG and clinical characteristics in heart failure subtypes

Admission time	Type	Dataset	AUC	Accuracy	Sensitivity	Specificity	PPV	NPV	F1 score	Brier score
First Admission	Systolic HF	Training Set	0.843 (0.816 - 0.870)	0.705 (0.687 - 0.723)	0.831 (0.795 - 0.868)	0.695 (0.678 - 0.712)	0.180 (0.165 - 0.194)	0.981 (0.977 - 0.985)	0.295 (0.273 - 0.317)	0.159 (0.139 - 0.179)
First Admission	Systolic HF	Validation Set	0.757 (0.724 - 0.791)	0.673 (0.652 - 0.693)	0.720 (0.677 - 0.763)	0.669 (0.647 - 0.691)	0.149 (0.132 - 0.166)	0.968 (0.962 - 0.973)	0.246 (0.222 - 0.270)	0.168 (0.146 - 0.189)

First Admission	Systolic HF	Test Set	0.721 (0.706 - 0.736)	0.689 (0.675 - 0.702)	0.636 (0.600 - 0.672)	0.693 (0.678 - 0.708)	0.139 (0.131 - 0.147)	0.961 (0.957 - 0.964)	0.228 (0.216 - 0.240)	0.165 (0.145 - 0.186)
First Admission	Diastolic HF	Training Set	0.811 (0.778 - 0.844)	0.748 (0.722 - 0.773)	0.730 (0.678 - 0.782)	0.749 (0.722 - 0.776)	0.152 (0.137 - 0.167)	0.978 (0.974 - 0.983)	0.437 (0.417 - 0.458)	0.172 (0.155 - 0.189)
First Admission	Diastolic HF	Validation Set	0.743 (0.727 - 0.759)	0.724 (0.704 - 0.743)	0.623 (0.602 - 0.644)	0.730 (0.710 - 0.750)	0.124 (0.104 - 0.144)	0.969 (0.964 - 0.974)	0.381 (0.358 - 0.404)	0.182 (0.164 - 0.200)
First Admission	Diastolic HF	Test Set	0.736 (0.726 - 0.746)	0.722 (0.698 - 0.746)	0.595 (0.544 - 0.645)	0.730 (0.702 - 0.758)	0.124 (0.118 - 0.130)	0.966 (0.963 - 0.969)	0.349 (0.335 - 0.362)	0.181 (0.164 - 0.198)
First Admission	Systolic and Diastolic HF	Training Set	0.842 (0.808 - 0.876)	0.684 (0.655 - 0.713)	0.871 (0.816 - 0.925)	0.669 (0.639 - 0.700)	0.173 (0.153 - 0.193)	0.985 (0.979 - 0.991)	0.251 (0.229 - 0.274)	0.149 (0.126 - 0.173)
First Admission	Systolic and Diastolic HF	Validation Set	0.770 (0.738 - 0.802)	0.652 (0.611 - 0.692)	0.772 (0.671 - 0.873)	0.642 (0.596 - 0.687)	0.147 (0.116 - 0.177)	0.974 (0.963 - 0.984)	0.206 (0.179 - 0.234)	0.155 (0.133 - 0.178)
First Admission	Systolic and Diastolic HF	Test Set	0.794 (0.770 - 0.818)	0.580 (0.535 - 0.624)	0.817 (0.756 - 0.878)	0.561 (0.513 - 0.609)	0.127 (0.112 - 0.142)	0.975 (0.967 - 0.983)	0.205 (0.196 - 0.214)	0.156 (0.134 - 0.178)

First Admission	Hypertensive HF	Training Set	0.842 (0.818 - 0.866)	0.763 (0.742 - 0.783)	0.782 (0.743 - 0.821)	0.761 (0.739 - 0.784)	0.192 (0.178 - 0.207)	0.980 (0.976 - 0.984)	0.371 (0.346 - 0.397)	0.164 (0.143 - 0.185)
First Admission	Hypertensive HF	Validation Set	0.773 (0.735 - 0.811)	0.734 (0.712 - 0.755)	0.683 (0.608 - 0.759)	0.738 (0.719 - 0.757)	0.158 (0.140 - 0.176)	0.969 (0.959 - 0.980)	0.313 (0.291 - 0.335)	0.171 (0.153 - 0.189)
First Admission	Hypertensive HF	Test Set	0.743 (0.730 - 0.755)	0.740 (0.707 - 0.772)	0.626 (0.574 - 0.677)	0.749 (0.714 - 0.783)	0.162 (0.141 - 0.183)	0.963 (0.958 - 0.968)	0.329 (0.318 - 0.340)	0.171 (0.152 - 0.190)
First Admission	Other HF	Training Set	0.827 (0.797 - 0.857)	0.725 (0.704 - 0.747)	0.783 (0.739 - 0.828)	0.722 (0.700 - 0.743)	0.162 (0.147 - 0.177)	0.980 (0.976 - 0.984)	0.288 (0.258 - 0.318)	0.164 (0.141 - 0.186)
First Admission	Other HF	Validation Set	0.746 (0.722 - 0.770)	0.699 (0.677 - 0.722)	0.677 (0.634 - 0.719)	0.701 (0.676 - 0.725)	0.134 (0.117 - 0.151)	0.970 (0.968 - 0.972)	0.246 (0.198 - 0.294)	0.170 (0.149 - 0.191)
First Admission	Other HF	Test Set	0.707 (0.704 - 0.711)	0.691 (0.672 - 0.710)	0.593 (0.581 - 0.605)	0.698 (0.677 - 0.718)	0.122 (0.115 - 0.129)	0.961 (0.959 - 0.962)	0.220 (0.196 - 0.243)	0.188 (0.166 - 0.209)
Last Admission	Systolic HF	Training Set	0.828 (0.806 - 0.851)	0.684 (0.668 - 0.700)	0.846 (0.814 - 0.878)	0.656 (0.641 - 0.672)	0.295 (0.280 - 0.310)	0.962 (0.954 - 0.970)	0.471 (0.451 - 0.491)	0.183 (0.165 - 0.201)

Last Admission	Systolic HF	Validation Set	0.746 (0.729 - 0.762)	0.650 (0.633 - 0.666)	0.742 (0.716 - 0.768)	0.634 (0.614 - 0.654)	0.257 (0.236 - 0.278)	0.935 (0.929 - 0.942)	0.402 (0.379 - 0.425)	0.197 (0.183 - 0.210)
Last Admission	Systolic HF	Test Set	0.723 (0.710 - 0.736)	0.654 (0.641 - 0.666)	0.681 (0.653 - 0.710)	0.649 (0.635 - 0.664)	0.234 (0.224 - 0.244)	0.928 (0.922 - 0.934)	0.300 (0.276 - 0.324)	0.214 (0.196 - 0.233)
Last Admission	Diastolic HF	Training Set	0.796 (0.767 - 0.826)	0.715 (0.689 - 0.741)	0.738 (0.693 - 0.784)	0.712 (0.682 - 0.742)	0.248 (0.229 - 0.268)	0.955 (0.948 - 0.962)	0.308 (0.290 - 0.327)	0.145 (0.123 - 0.168)
Last Admission	Diastolic HF	Validation Set	0.717 (0.698 - 0.735)	0.695 (0.678 - 0.711)	0.612 (0.585 - 0.639)	0.705 (0.687 - 0.724)	0.211 (0.192 - 0.230)	0.934 (0.928 - 0.940)	0.257 (0.230 - 0.283)	0.153 (0.128 - 0.179)
Last Admission	Diastolic HF	Test Set	0.732 (0.723 - 0.741)	0.691 (0.673 - 0.708)	0.623 (0.562 - 0.683)	0.700 (0.672 - 0.728)	0.224 (0.219 - 0.229)	0.931 (0.922 - 0.939)	0.257 (0.227 - 0.286)	0.152 (0.131 - 0.173)
Last Admission	Systolic and Diastolic HF	Training Set	0.802 (0.768 - 0.835)	0.657 (0.633 - 0.681)	0.852 (0.804 - 0.901)	0.615 (0.587 - 0.642)	0.325 (0.312 - 0.339)	0.950 (0.933 - 0.967)	0.375 (0.353 - 0.397)	0.158 (0.137 - 0.179)
Last Admission	Systolic and Diastolic HF	Validation Set	0.709 (0.665 - 0.754)	0.600 (0.580 - 0.621)	0.763 (0.660 - 0.866)	0.567 (0.532 - 0.602)	0.275 (0.253 - 0.298)	0.915 (0.876 - 0.953)	0.316 (0.295 - 0.337)	0.167 (0.144 - 0.190)

Last Admission	Systolic and Diastolic HF	Test Set	0.651 (0.622 - 0.679)	0.523 (0.503 - 0.544)	0.708 (0.642 - 0.773)	0.492 (0.469 - 0.515)	0.190 (0.176 - 0.205)	0.909 (0.891 - 0.928)	0.329 (0.311 - 0.347)	0.165 (0.147 - 0.184)
Last Admission	Hypertensive HF	Training Set	0.823 (0.799 - 0.847)	0.733 (0.707 - 0.759)	0.773 (0.732 - 0.814)	0.729 (0.699 - 0.759)	0.248 (0.229 - 0.267)	0.965 (0.959 - 0.972)	0.268 (0.245 - 0.291)	0.154 (0.132 - 0.176)
Last Admission	Hypertensive HF	Validation Set	0.736 (0.700 - 0.772)	0.707 (0.683 - 0.731)	0.658 (0.588 - 0.727)	0.713 (0.688 - 0.738)	0.208 (0.196 - 0.221)	0.947 (0.933 - 0.961)	0.224 (0.199 - 0.249)	0.161 (0.139 - 0.183)
Last Admission	Hypertensive HF	Test Set	0.735 (0.721 - 0.750)	0.712 (0.682 - 0.741)	0.638 (0.586 - 0.691)	0.721 (0.683 - 0.758)	0.222 (0.207 - 0.238)	0.942 (0.936 - 0.948)	0.202 (0.192 - 0.211)	0.164 (0.144 - 0.185)
Last Admission	Other HF	Training Set	0.811 (0.786 - 0.837)	0.692 (0.671 - 0.712)	0.801 (0.766 - 0.837)	0.673 (0.651 - 0.695)	0.294 (0.276 - 0.312)	0.952 (0.944 - 0.961)	0.430 (0.406 - 0.453)	0.171 (0.153 - 0.189)
Last Admission	Other HF	Validation Set	0.733 (0.722 - 0.744)	0.664 (0.645 - 0.684)	0.696 (0.670 - 0.721)	0.659 (0.633 - 0.685)	0.257 (0.244 - 0.270)	0.928 (0.924 - 0.931)	0.375 (0.362 - 0.389)	0.179 (0.162 - 0.196)
Last Admission	Other HF	Test Set	0.702 (0.698 - 0.706)	0.652 (0.638 - 0.666)	0.638 (0.605 - 0.670)	0.654 (0.633 - 0.675)	0.235 (0.231 - 0.239)	0.916 (0.911 - 0.921)	0.343 (0.338 - 0.348)	0.183 (0.166 - 0.200)

AUC – Area Under the Curve, PPV – Positive Predictive Value, NPV – Negative Predictive Value, ECG – Electrocardiogram, HF – heart failure.

Table S8. DiscrimiTRIPOD Checklist: Prediction Model Development

Section/Topic	Item	Checklist Item	Page
**Title and abstract**			
Title	1	Identify the study as developing and/or validating a multivariable prediction model, the target population, and the outcome to be predicted.	1
Abstract	2	Provide a summary of objectives, study design, setting, participants, sample size, predictors, outcome, statistical analysis, results, and conclusions.	1
**Introduction**			
Background and objectives	3a	Explain the medical context (including whether diagnostic or prognostic) and rationale for developing or validating the multivariable prediction model, including references to existing models.	2
	3b	Specify the objectives, including whether the study describes the development or validation of the model or both.	3
**Methods**			
Source of data	4a	Describe the study design or source of data (e.g., randomized trial, cohort, or registry data), separately for the development and validation data sets, if applicable.	3
	4b	Specify the key study dates, including start of accrual; end of accrual; and, if applicable, end of follow-up.	3
Participants	5a	Specify key elements of the study setting (e.g., primary care, secondary care, general population) including number and location of centres.	3
	5b	Describe eligibility criteria for participants.	4
	5c	Give details of treatments received, if relevant.	n/a
Outcome	6a	Clearly define the outcome that is predicted by the prediction model, including how and when assessed.	5
	6b	Report any actions to blind assessment of the outcome to be predicted.	n/a
Predictors	7a	Clearly define all predictors used in developing or validating the multivariable prediction model, including how and when they were measured.	5
	7b	Report any actions to blind assessment of predictors for the outcome and other predictors.	n/a
Sample size	8	Explain how the study size was arrived at.	4
Missing data	9	Describe how missing data were handled (e.g., complete-case analysis, single imputation, multiple imputation) with details of any imputation method.	4
Statistical analysis methods	10a	Describe how predictors were handled in the analyses.	5
	10b	Specify type of model, all model-building procedures (including any predictor selection), and method for internal validation.	Supplementary Document

	10d	Specify all measures used to assess model performance and, if relevant, to compare multiple models.	6
Risk groups	11	Provide details on how risk groups were created, if done.	Supplementary Document
**Results**			
Participants	13a	Describe the flow of participants through the study, including the number of participants with and without the outcome and, if applicable, a summary of the follow-up time. A diagram may be helpful.	6
	13b	Describe the characteristics of the participants (basic demographics, clinical features, available predictors), including the number of participants with missing data for predictors and outcome.	7
Model development	14a	Specify the number of participants and outcome events in each analysis.	7
	14b	If done, report the unadjusted association between each candidate predictor and outcome.	n/a
Model specification	15a	Present the full prediction model to allow predictions for individuals (i.e., all regression coefficients, and model intercept or baseline survival at a given time point).	Supplementary Document
	15b	Explain how to the use the prediction model.	Supplementary Document
Model performance	16	Report performance measures (with CIs) for the prediction model.	8
**Discussion**			
Limitations	18	Discuss any limitations of the study (such as nonrepresentative sample, few events per predictor, missing data).	12
Interpretation	19b	Give an overall interpretation of the results, considering objectives, limitations, and results from similar studies, and other relevant evidence.	11
Implications	20	Discuss the potential clinical use of the model and implications for future research.	12
**Other information**			
Supplementary information	21	Provide information about the availability of supplementary resources, such as study protocol, Web calculator, and data sets.	Supplementary Document
Funding	22	Give the source of funding and the role of the funders for the present study.	13
